# Supplementary material for: Photoelectrochemical Response Enhancement for Metallofullerene-[12]Cycloparaphenylene Supramolecular Complexes
Source: Nanomaterials (Basel). 2022 Apr 20;12(9):1408. doi: 10.3390/nano12091408 (PMC9104806; doi:10.3390/nano12091408)
Supplement: Supplementary file 1 [file nanomaterials-12-01408-s001.zip › nanomaterials-1680889-supplementary.pdf]

nanomaterials

Supplementary Materials of

Photoelectrochemical Response Enhancement for Metallofullerene-[12] Cycloparaphenylene Supramolecular Complexes

Jie Zhang [1,2,3], Ling Qiu [1], Linshan Liu [1], Yang Liu [1], Peng Cui [4,*], Fang Wang [5] and Zhuxia Zhang [1,*]

[1] College of Chemistry, Taiyuan University of Technology, Taiyuan 030024, China; ql7222785@163.com (L.Q.); l_lynne33@163.com (L.L.); liuliuyang258@163.com (Y.L.)
[2] Beijing National Laboratory for Molecular Sciences, Key Laboratory of Molecular Nanostructure and Nanotechnology, Institute of Chemistry, Chinese Academy of Sciences, Beijing100190, China; zhangjie654@iccas.ac.cn
[3] University of Chinese Academy of Sciences, Beijing 100049, China
[4] School of Information, Guizhou University of Finance and Economics, Guiyang 550025, China
[5] Collaborative Innovation Center for Shanxi Advanced Permanent Magnetic Materials and Technology, Linfen 041004, China; wangfanghc@sxnu.edu.cn
* Correspondence: pcui@ustc.edu.cn (P.C.); zhangzhuxia@tyut.edu.cn (Z.Z.)

MALDI-TOF

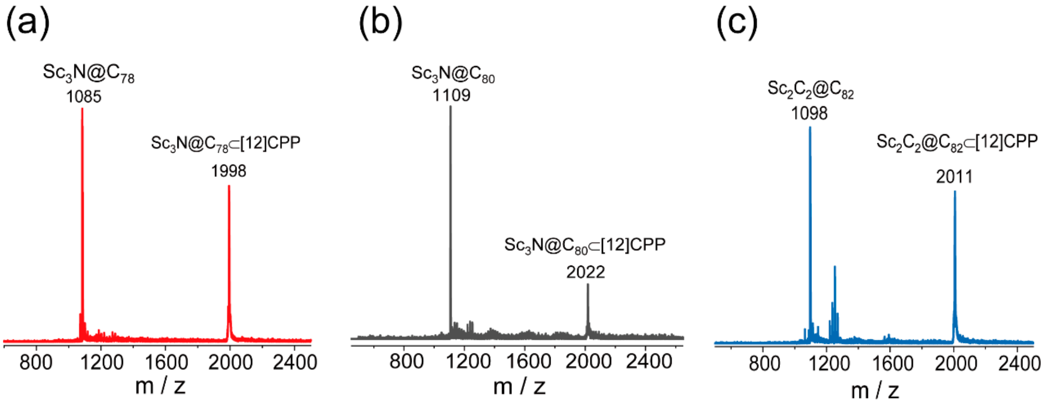

Figure S1. Corresponding MALDI-TOF mass spectra of prepared (a) $Sc_3N@C_{78}$@[12]CPP, (b) $Sc_3N@C_{80}$@[12]CPP, and (c) $Sc_2C_2@C_{82}$@[12]CPP, respectively.

UV-Vis-NIR Spectrum

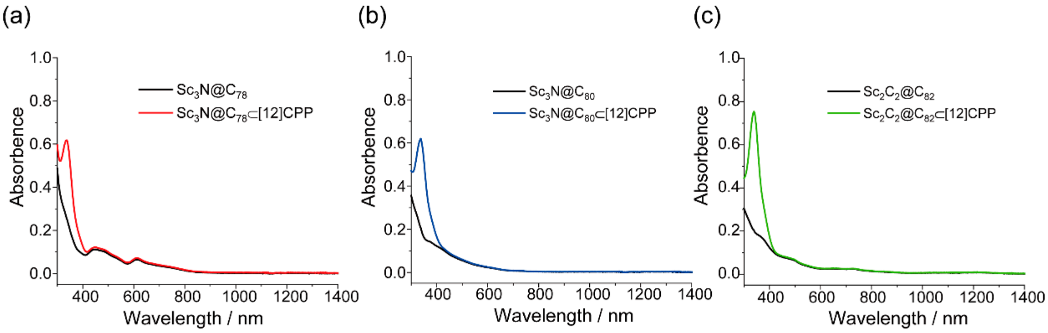

Figure S2. UV-Vis-NIR absorption spectra of (a) $Sc_3N@C_{78}$ and $Sc_3N@C_{78}\subset$[12]CPP, (b) $Sc_3N@C_{80}$ and $Sc_3N@C_{80}\subset$[12]CPP, and (c) $Sc_2C_2@C_{82}$ and $Sc_2C_2@C_{82}\subset$[12]CPP, respectively.

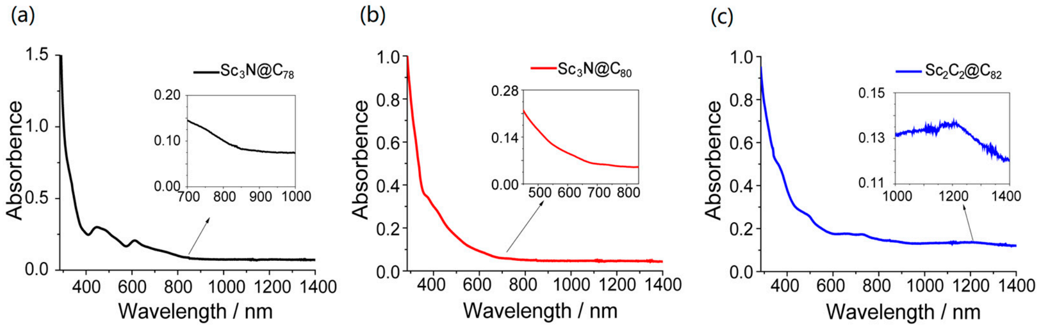

Figure S3. UV-vis-NIR absorption spectra of (a) $Sc_3N@C_{78}$, (b) $Sc_3N@C_{80}$ and (c) $Sc_2C_2@C_{82}$, respectively. The insets show the enlarged spectra around onset wavelength.

Bonding Energies

Bonding energies were calculated at the level of B3LYP/6-31G* and realized by DFT [31]. The Gaussian 16 (A.03) program package was employed for all quantum chemistry calculations.

Table S1. The optimized energies of complex EMFs⊂[12]CPP, EMFs and [12]CPP ($Kcal \cdot mol^{-1}$).

Energy	EMFs+[12]CPP	EMFs	[12]CPP	Bonding Energy(ΔE)
$Sc_2C_2@C_{82}$⊂[12]CPP	−4703407.9006	−2963394.0343	−1739965.4938	−48.3725
$Sc_3N@C_{78}$⊂[12]CPP	−5071731.4772	−3331721.9132	−1739965.4938	−44.0702
$Sc_3N@C_{80}$⊂[12]CPP	−5119591.0014	−3379581.2244	−1739965.4938	−44.2832

Bonding energies ΔE were evaluated in the following equation:

$$\Delta E = E(EMFs + [12]CPP) - E(EMFs) - E([12]CPP)$$

where E(EMFs + [12]CPP), E(EMFs) and E([12]CPP) are total energies of each compound.

SEM Images

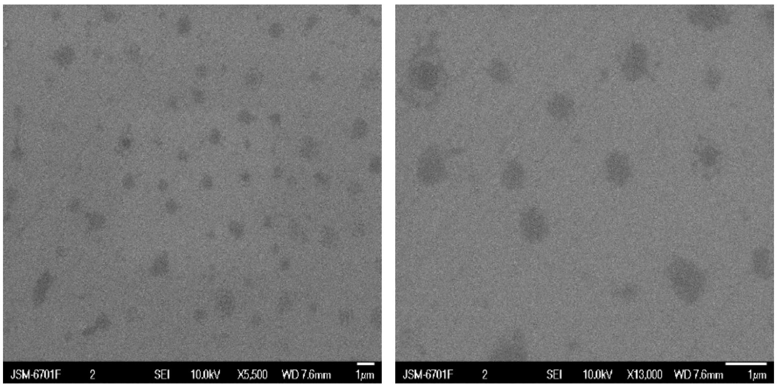

Figure S4. SEM image of $Sc_2C_2@C_{82}$.

Photocurrent Response of Monomer

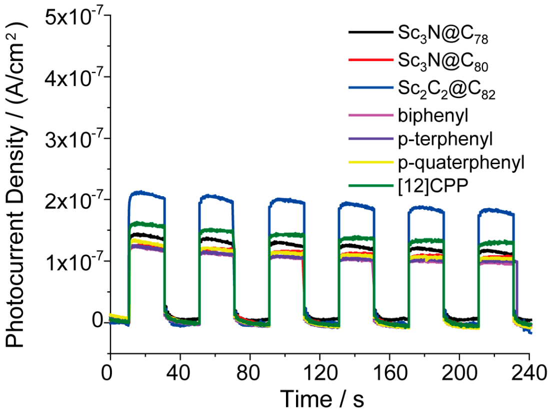

Figure S5. Photocurrent response intensity of metallofullerenes, biphenyl, p-terphenyl, p-quaterphenyl and [12]CPP on FTO glass.

UV-Vis-NIR spectra of $Sc_3N@C_{78}$, $Sc_3N@C_{80}$ and $Sc_2C_2@C_{82}$ with biphenyl (2.4×10^{-4} M), p-terphenyl (1.6×10^{-4} M), p-quaterphenyl (1.2×10^{-4} M) and [12]CPP. The different concentrations were employed to ensure that to ensure that they have the same number of benzene rings with [12]CPP.

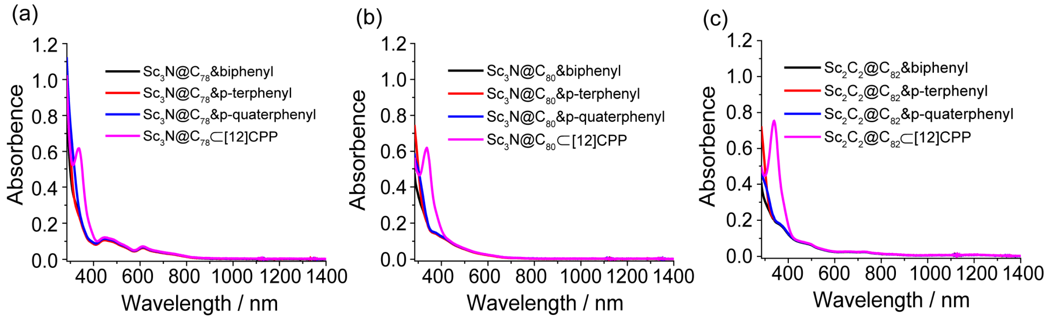

Figure S6. UV-Vis-NIR spectra of (a)$Sc_3N@C_{78}$, (b) $Sc_3N@C_{80}$ and (c) $Sc_2C_2@C_{82}$ with biphenyl, p-terphenyl, p-quaterphenyl and [12]CPP, respectively.
